# Supplementary material for: The Prevalence of Virulence Factor Genes among Carbapenem-Non-Susceptible Acinetobacter baumannii Clinical Strains and Their Usefulness as Potential Molecular Biomarkers of Infection
Source: Diagnostics (Basel). 2023 Mar 8;13(6):1036. doi: 10.3390/diagnostics13061036 (PMC10047099; doi:10.3390/diagnostics13061036)
Supplement: Supplementary file 1 [file diagnostics-13-01036-s001.zip › diagnostics-2167281-supplementary.pdf]

Table S1. The origin of *Acinetobacter baumannii* (*n* = 100) clinical strains used in the study (units).

Clinic/Unit	Number (%) of strains
Anesthesiology and Intensive Care	63 (63.0)
Liver and General Surgery	11 (11.0)
Cardiology	6 (6.0)
Nephrology, Hypertension and Internal Medicine	5 (5.0)
Vascular Surgery and Angiology	3 (3.0)
Geriatrics	3 (3.0)
Endocrinology and Diabetology	2 (2.0)
Anaesthesiology and Intensive Care with Cardiac Anaesthesiology Division	2 (2.0)
Vascular Surgery	2 (2.0)
Rehabilitation	1 (1.0)
Cardiac Surgery	1 (1.0)
Chronic Wounds Care	1 (1.0)

Table S2. The occurrence of bla_{OXA} genes in *A. baumannii* with respect to strains' origin.

Clinical material	Number (%) of strains	$bla_{\mathrm{OXA-40}}$ (%)	$bla_{\mathrm{OXA-23}}$ (%)
Bronchoalveolar lavage	41 (41.0)	21 (51.2)	19 (46.3)
Wound swabs	25 (25.0)	18 (72.0)	6 (24.0)
Blood	10 (10.0)	7 (70.0)	3 (30.0)
Urine	6 (6.0)	4 (66.7)	1 (16.7)
Peritoneal swabs	3 (3.0)	3 (100.0)	0 (0.0)
Pus	3 (3.0)	2 (66.7)	1 (33.3)
Vascular catheter	2 (2.0)	1 (50.0)	1 (50.0)
Tissue	2 (2.0)	2 (100.0)	0 (0.0)
Respiratory tract secretion	2 (2.0)	1 (50.0)	1 (50.0)
Peritoneal fluid	1 (1.0)	1 (100.0)	0 (0.0)
Sputum	1 (1.0)	0 (0.0)	1 (100.0)
Cerebrospinal fluid	1 (1.0)	1 (100.0)	0 (0.0)
Pleural fluid	1 (1.0)	1 (100.0)	0 (0.0)
Tracheal swab	1 (1.0)	0 (0.0)	1 (100.0)
Granulation tissue	1 (1.0)	0 (0.0)	1 (100.0)

Table S3. The distribution of virulence factors genes among the reference *Acinetobacter baumannii* strains.

	bap	*surA1*	*basD*	*bauA*	*pld*	*omp33-36*
DSM 102930	+	+	+	-	+	+
DSM 30008	+	+	+	+	+	-

(+) – presence of a particular gene; (-) – absence of a particular gene; DSMZ - Deutsche Sammlung von Mikroorganismen und Zellkulturen, Germany

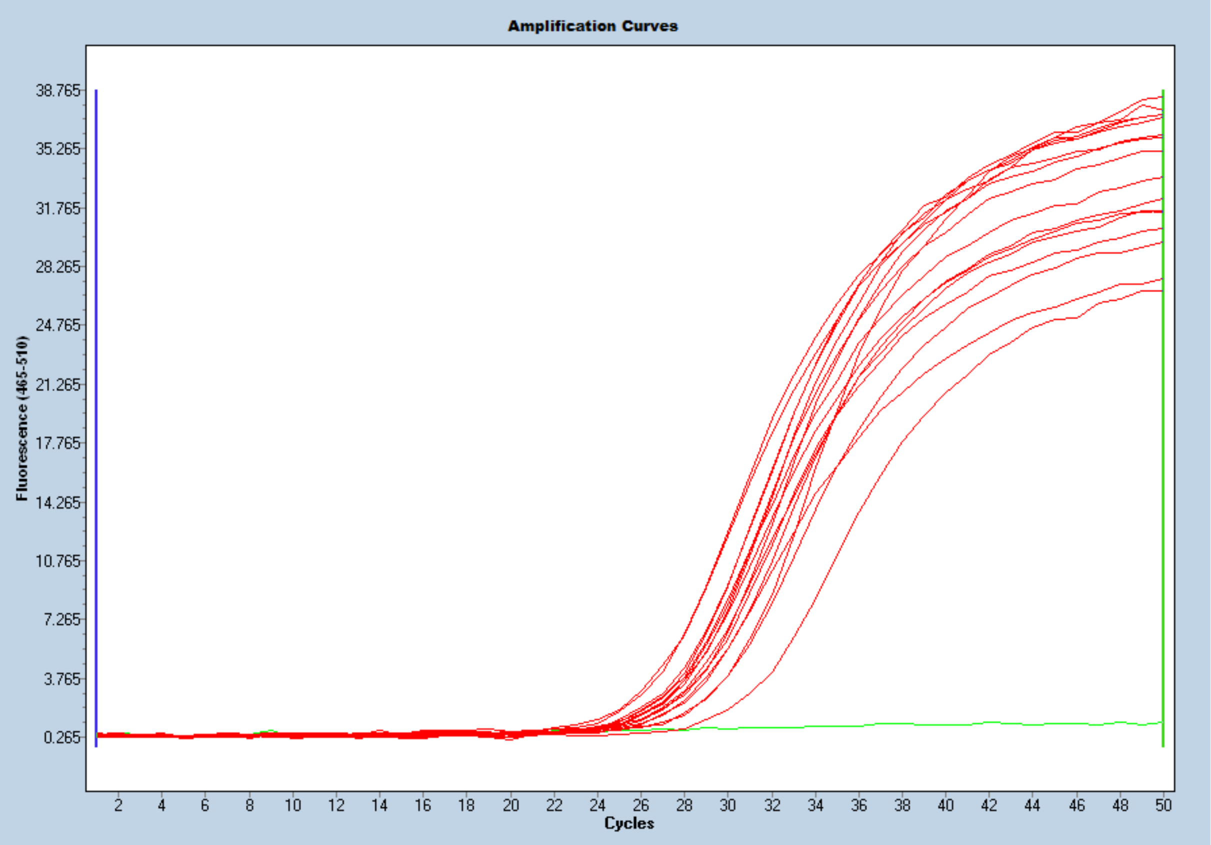

Figure S1. Picture of amplification curves showing results of real-time PCR for the *bap* gene detection – LightCycler 480 II (Roche, Basel, Switzerland); red lines – positive results, including positive control; green line – negative control.

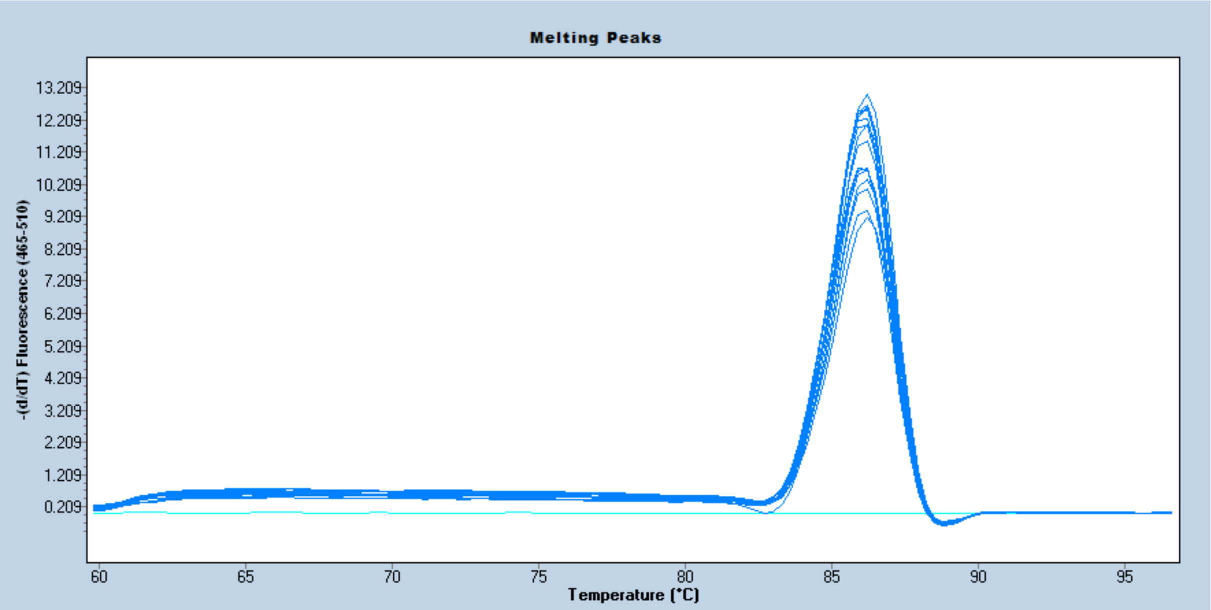

Figure S2. An example of picture showing melting peaks of the real-time PCR product specificity for the *bap* gene – LightCycler 480 II (Roche, Basel, Switzerland); dark blue lines – positive results, including positive control, showing specificity of the amplified product; light blue line – negative control.

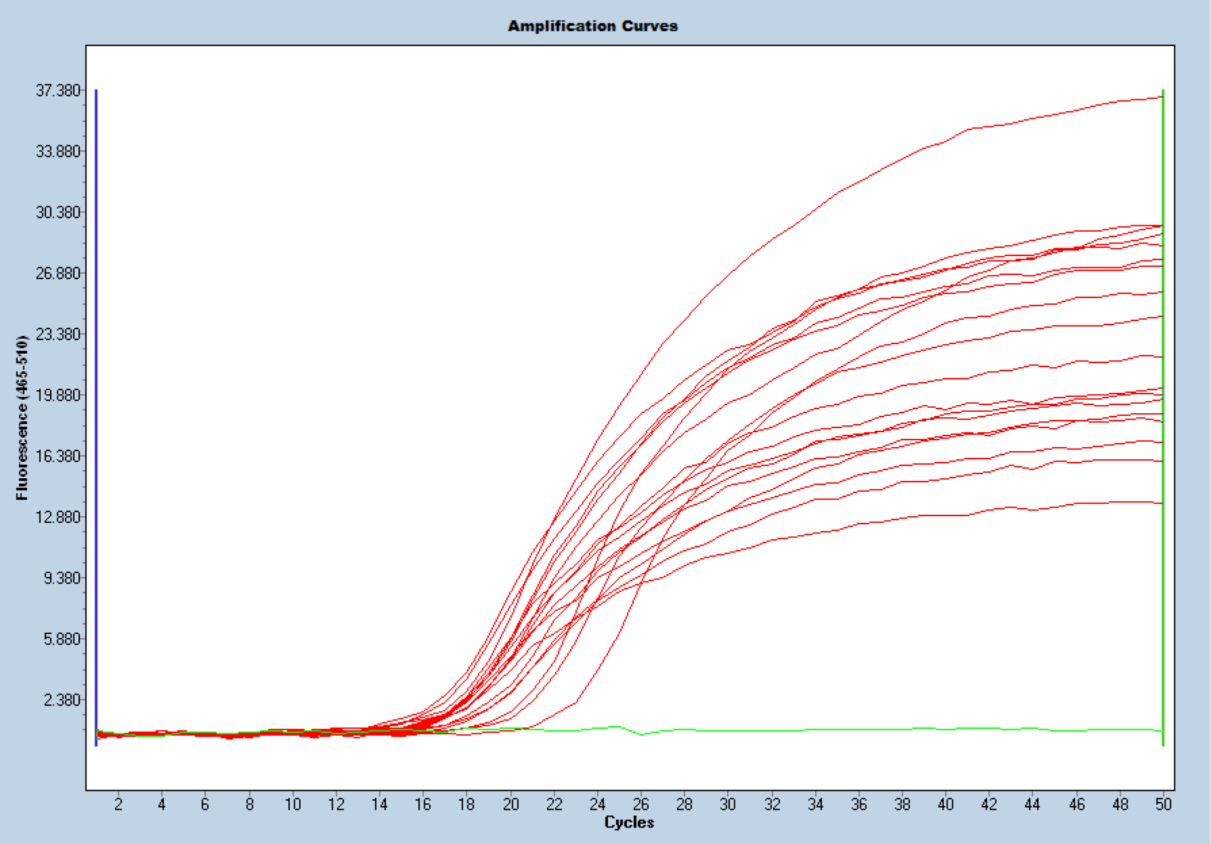

Figure S3. Picture of amplification curves showing results of real-time PCR for the *surA1* gene detection – LightCycler 480 II (Roche, Basel, Switzerland); red lines – positive results, including positive control; green line – negative control.

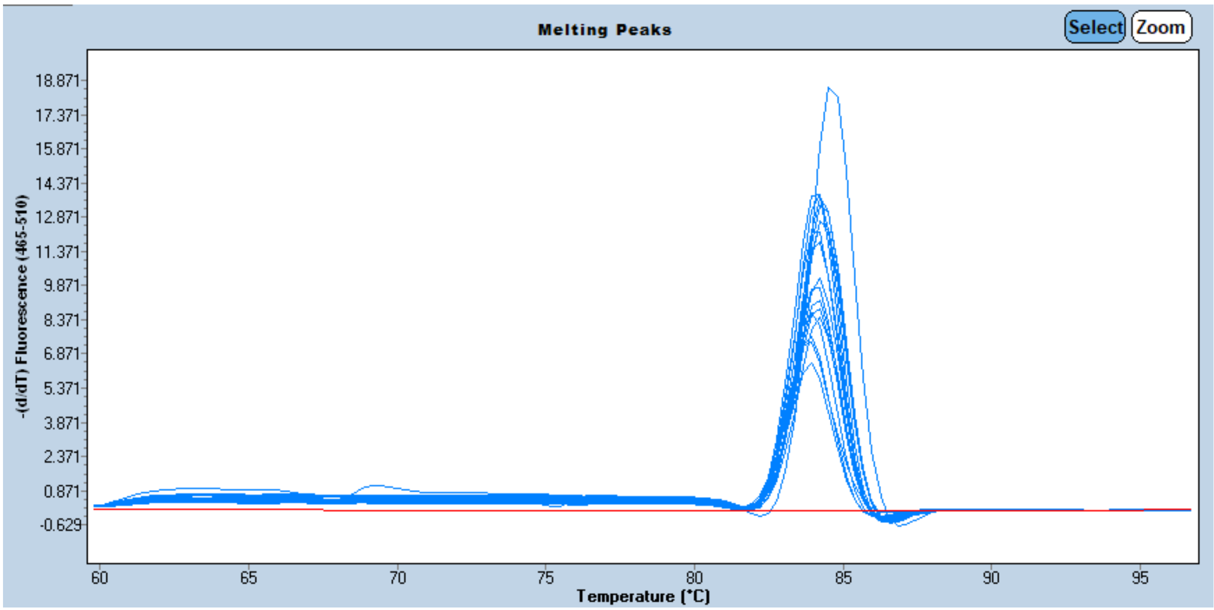

Figure S4. An example of picture showing melting peaks of the real-time PCR product specificity for the *surA1* gene – LightCycler 480 II (Roche, Basel, Switzerland); blue lines – positive results, including positive control, showing specificity of the amplified product; red line – negative control.

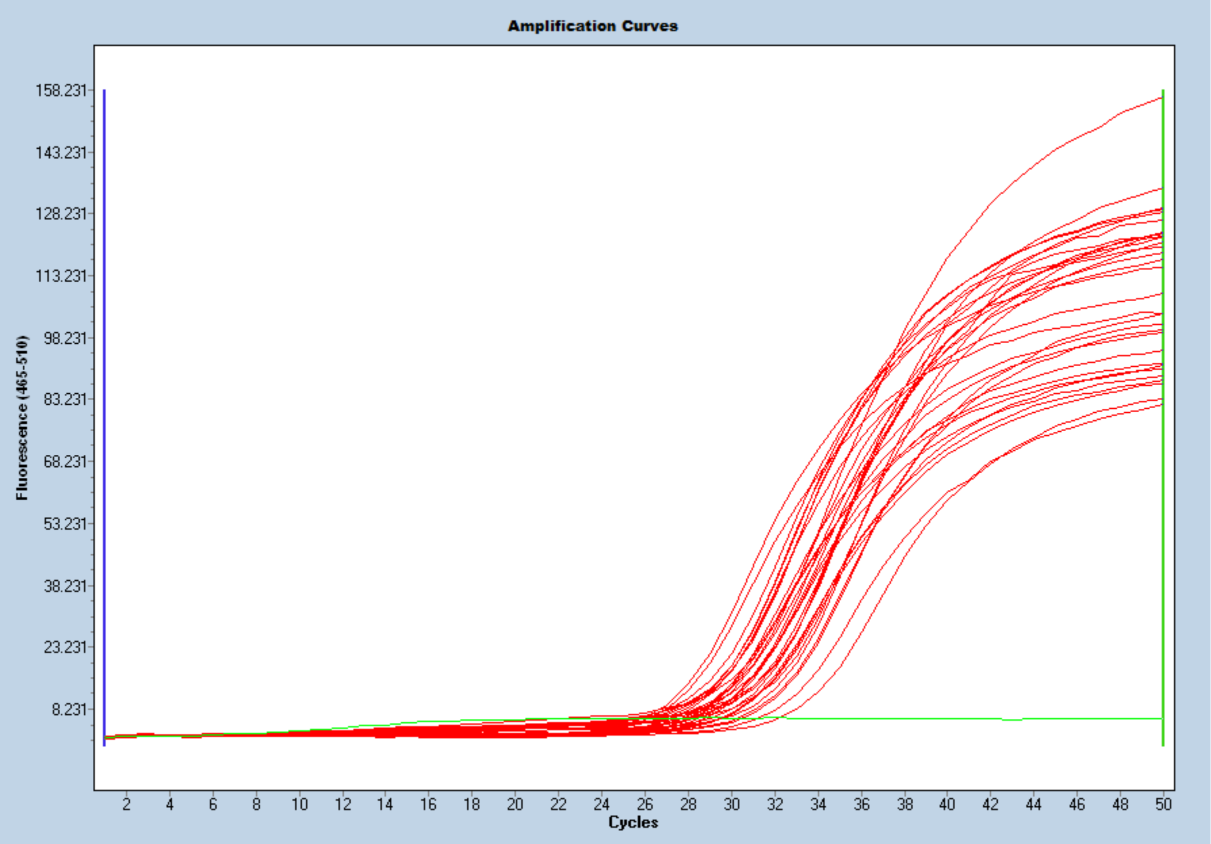

Figure S5. Picture of amplification curves showing results of real-time PCR for the *basD* gene detection – LightCycler 480 II (Roche, Basel, Switzerland); red lines – positive results, including positive control; green line – negative control.

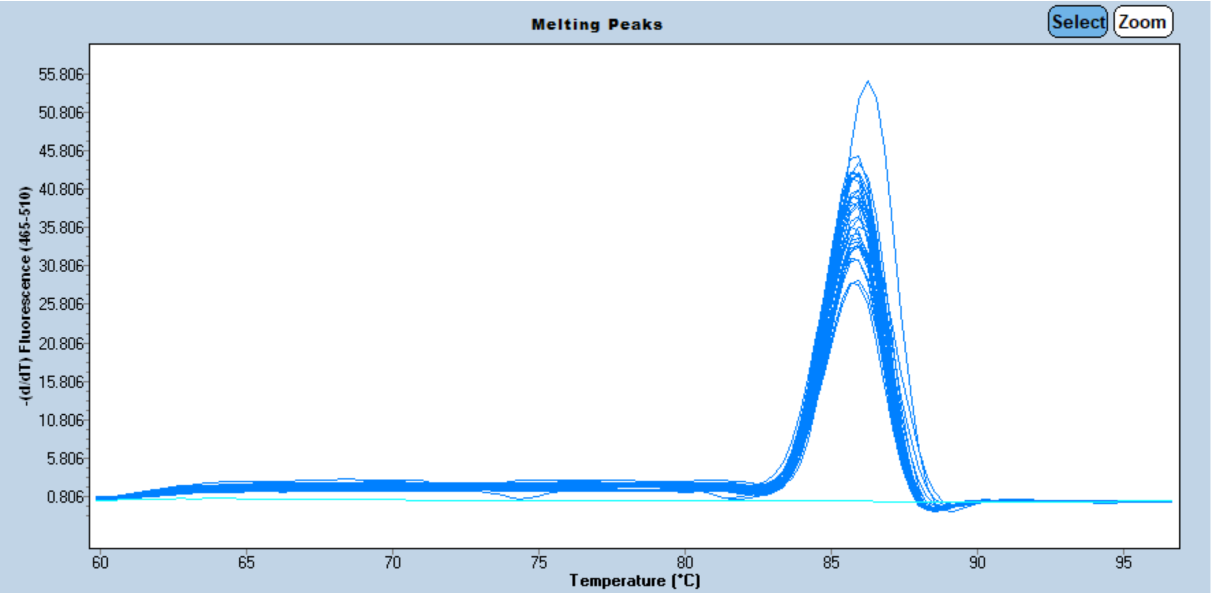

Figure S6. An example of picture showing melting peaks of the real-time PCR product specificity for the *basD* gene – LightCycler 480 II (Roche, Basel, Switzerland); dark blue lines – positive results, including positive control, showing specificity of the amplified product; light blue line – negative control.

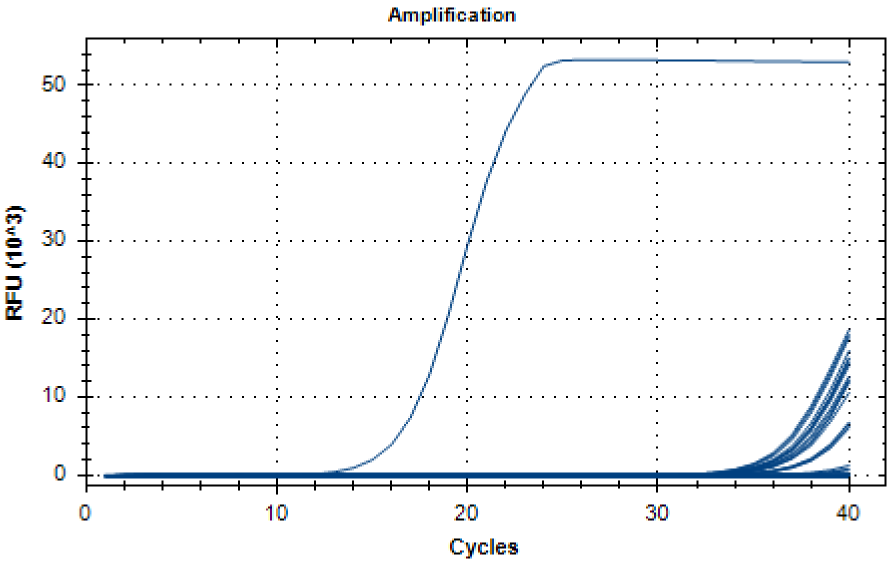

Figure S7. Picture of amplification curves showing results of real-time PCR for the *bauA* gene detection – CFX OPUS 96 (Bio-Rad, Hercules, United States); an amplification curve with Cycle threshold (Ct) around 16th cycle – positive control; the remaining curves – negative results.

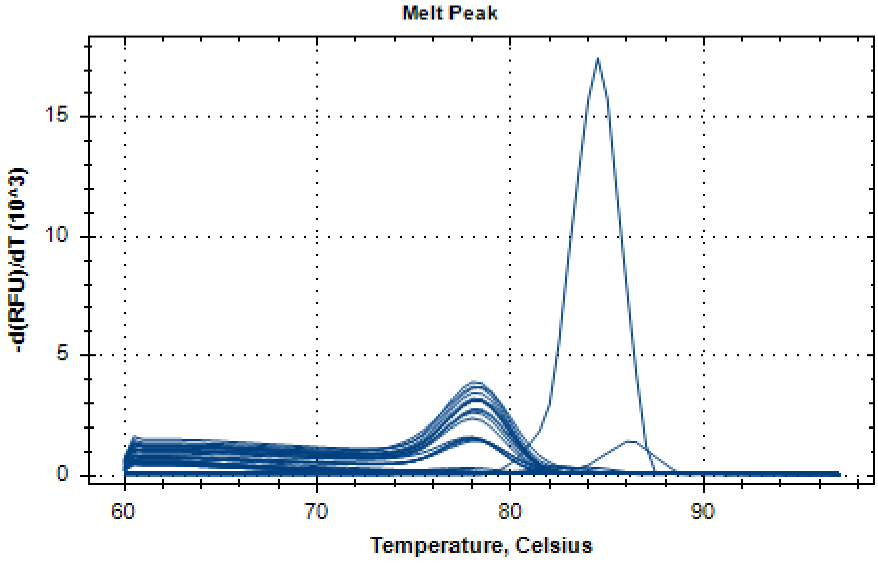

Figure S8. An example of picture showing melting peaks of the real-time PCR product specificity for the *bauA* gene – CFX OPUS 96 (Bio-Rad, Hercules, United States), the peak with melting point at 84.5°C – positive control showing specificity of the amplified product, the remaining lines – negative results.

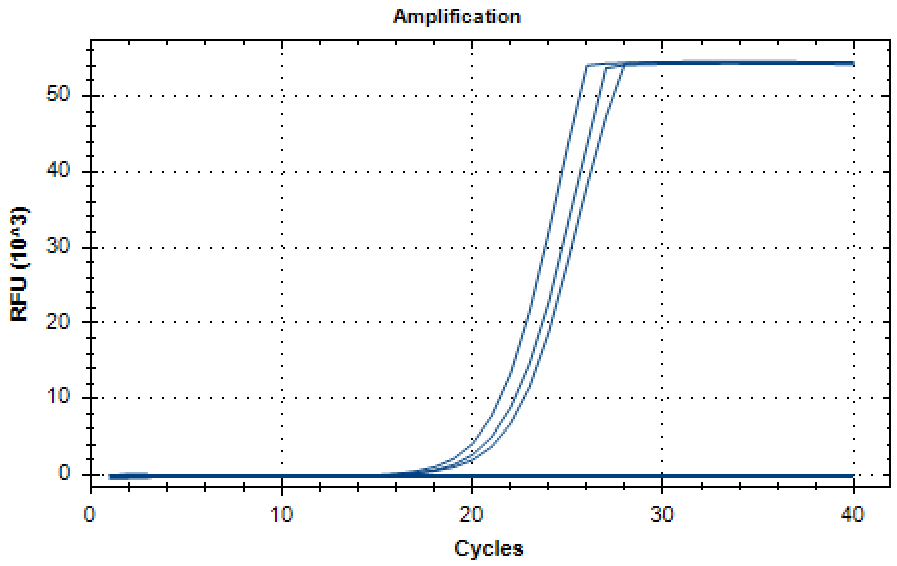

Figure S9. Picture of amplification curves showing results of real-time PCR for the *pld* gene detection – CFX OPUS 96 (Bio-Rad, Hercules, United States); amplification curves with Ct around 21st cycle – positive results, including positive control; the remaining curves – negative results.

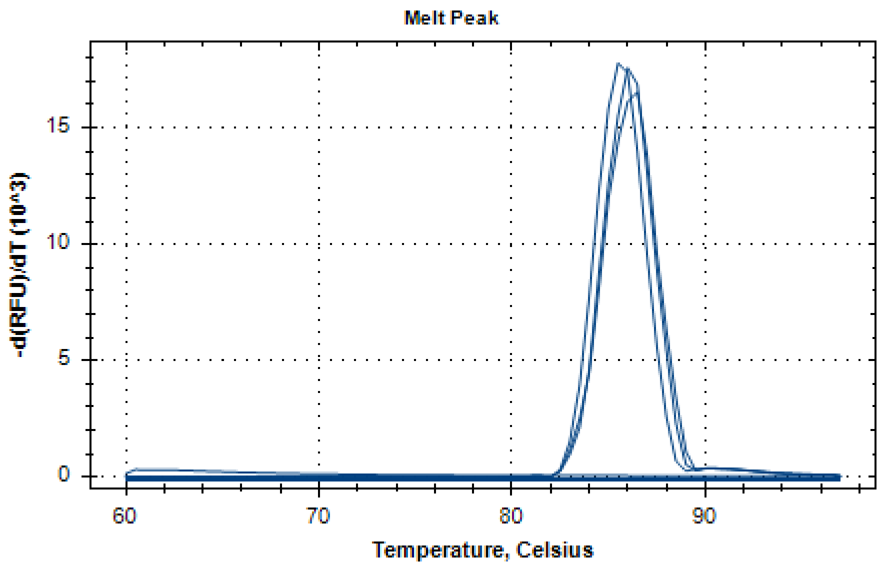

Figure S10. An example of picture showing melting peaks of the real-time PCR product specificity for the *pld* gene – CFX OPUS 96 (Bio-Rad, Hercules, United States); the peaks with melting point at 86°C – positive results showing specificity of the amplified product, the remaining lines – negative results.

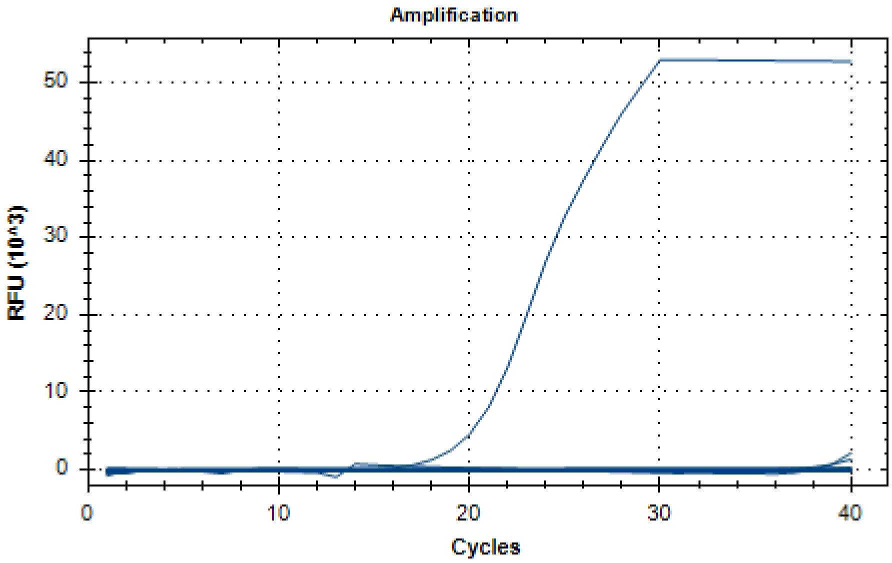

Figure S11. Picture of amplification curves showing results of real-time PCR for the *omp33-36* gene detection – CFX OPUS 96 (Bio-Rad, Hercules, United States); an amplification curve with Ct around 21st cycle – positive control; the remaining curves – negative results.

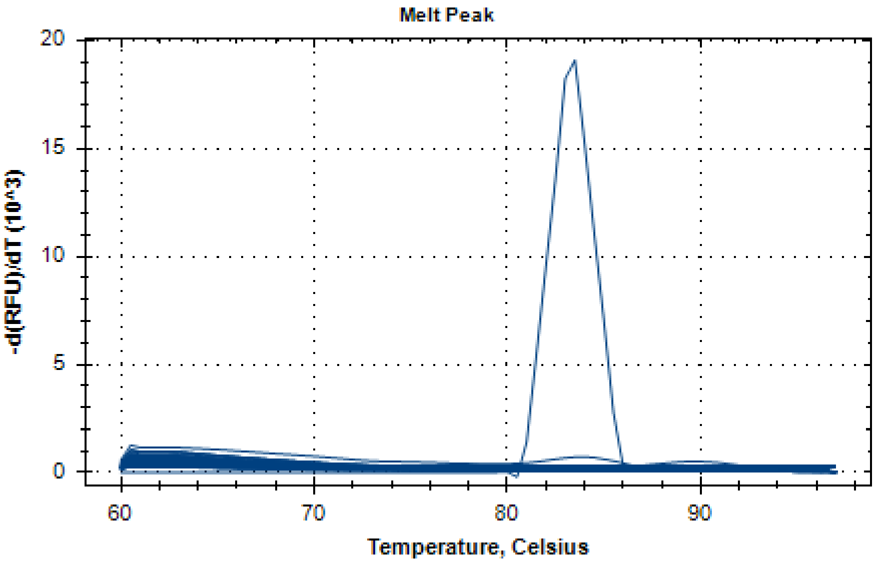

Figure S12. An example of picture showing melting peaks of the real-time PCR product specificity for the *omp33-36* gene – CFX OPUS 96 (Bio-Rad, Hercules, United States); the peak with melting point at 83.5°C – positive control showing specificity of the amplified product, the remaining lines – negative results.

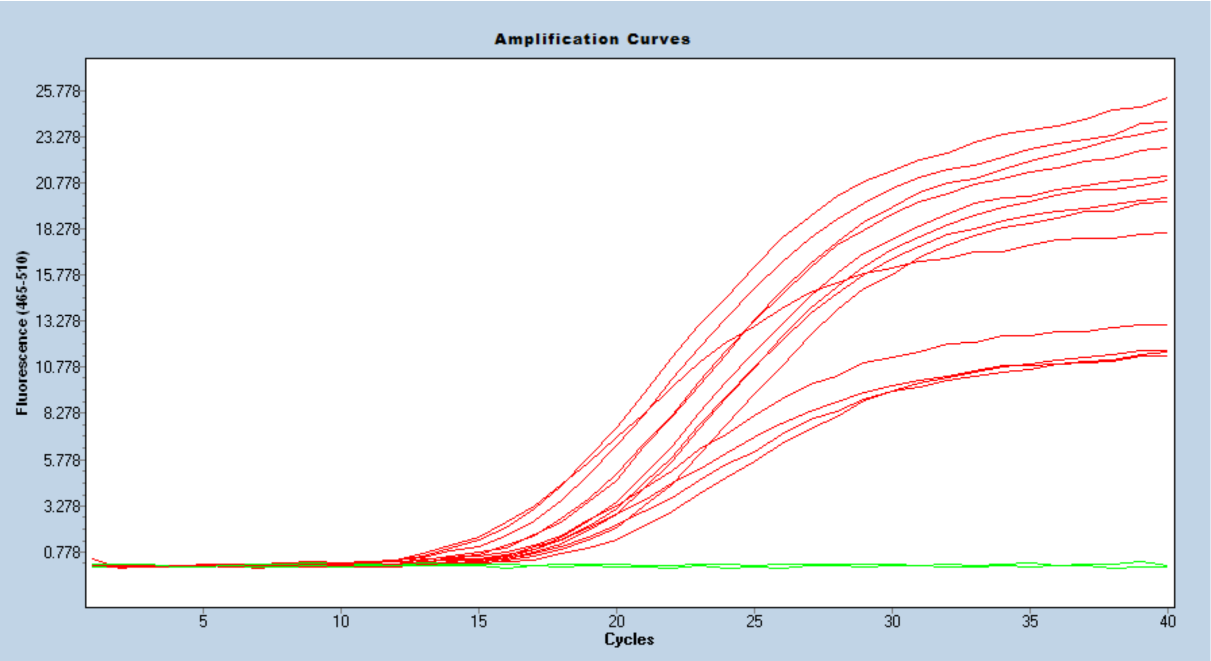

Figure S13. Picture of amplification curves showing results of real-time PCR for the *bla*$_{OXA-40}$ gene detection – LightCycler 480 II (Roche, Basel, Switzerland); red lines – positive results, including positive control; green lines – negative results.

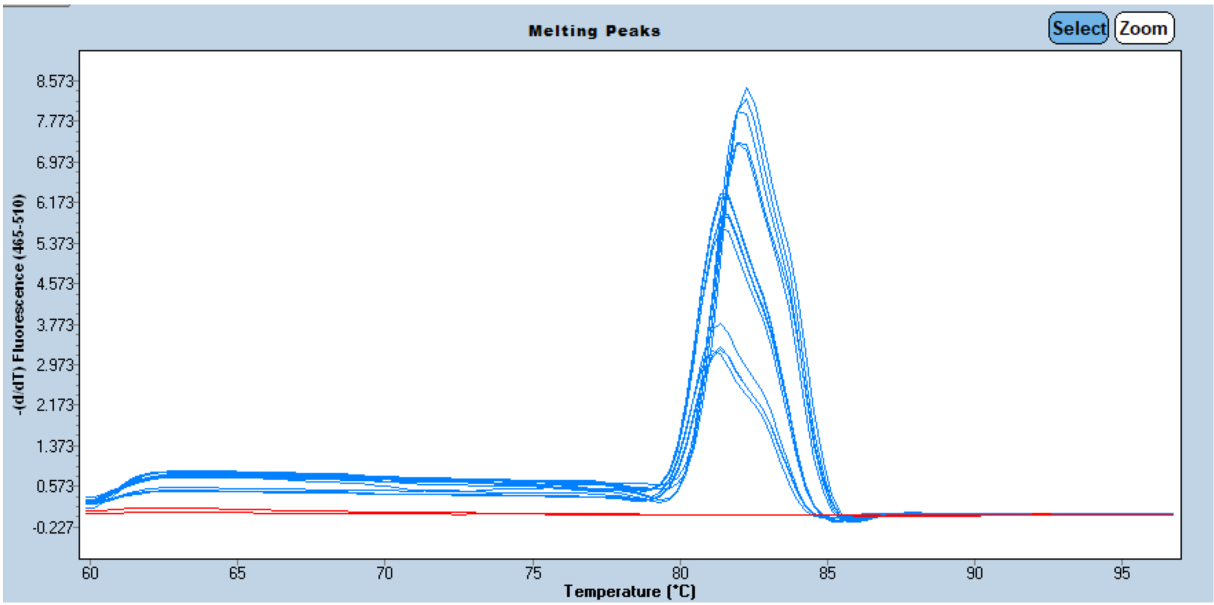

Figure S14. An example of melting peaks picture showing specificity of the real-time PCR product for the *bla*$_{OXA-40}$ gene – LightCycler 480 II (Roche, Basel, Switzerland); blue lines – positive results, including positive control, showing specificity of the amplified product; red lines – negative results.

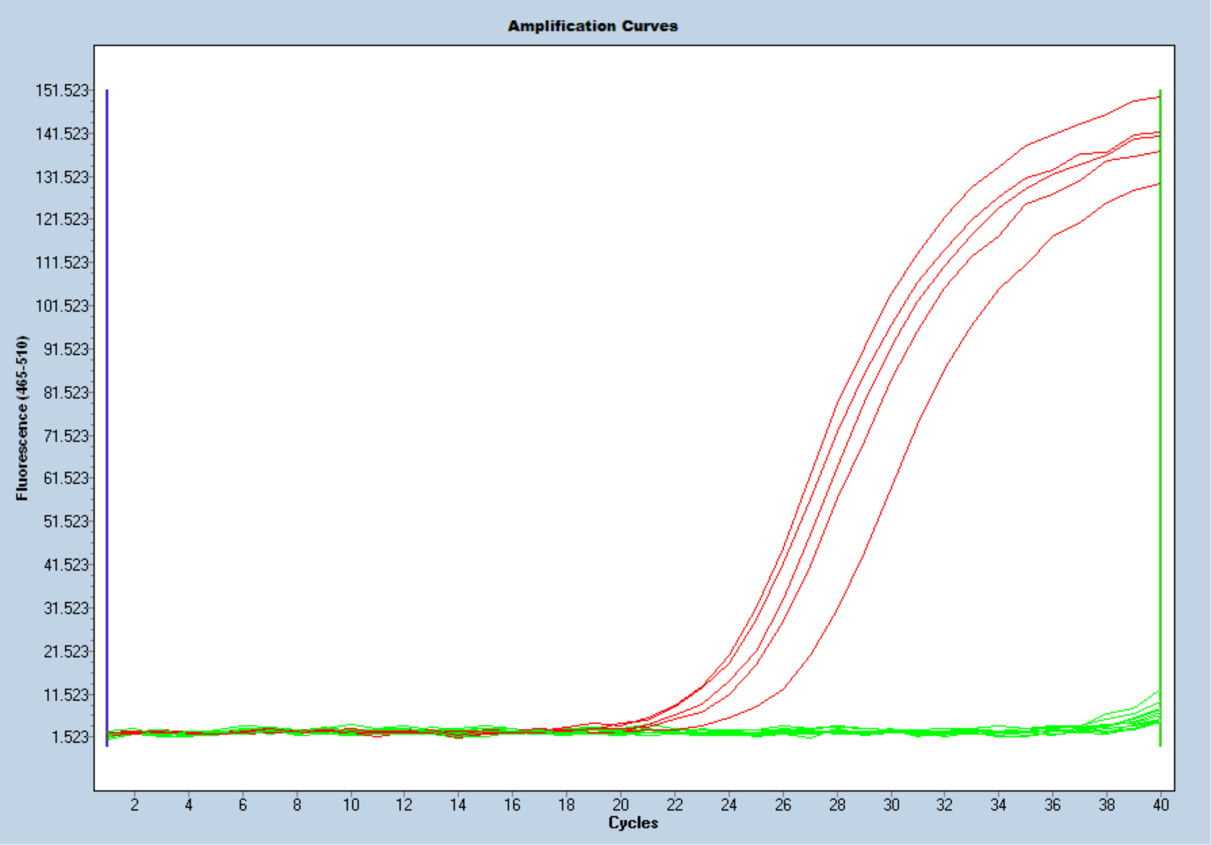

Figure S15. Picture of amplification curves showing results of real-time PCR for the *bla*$_{OXA-23}$ gene detection – LightCycler 480 II (Roche, Basel, Switzerland); red lines – positive results, including positive control; green lines – negative results.

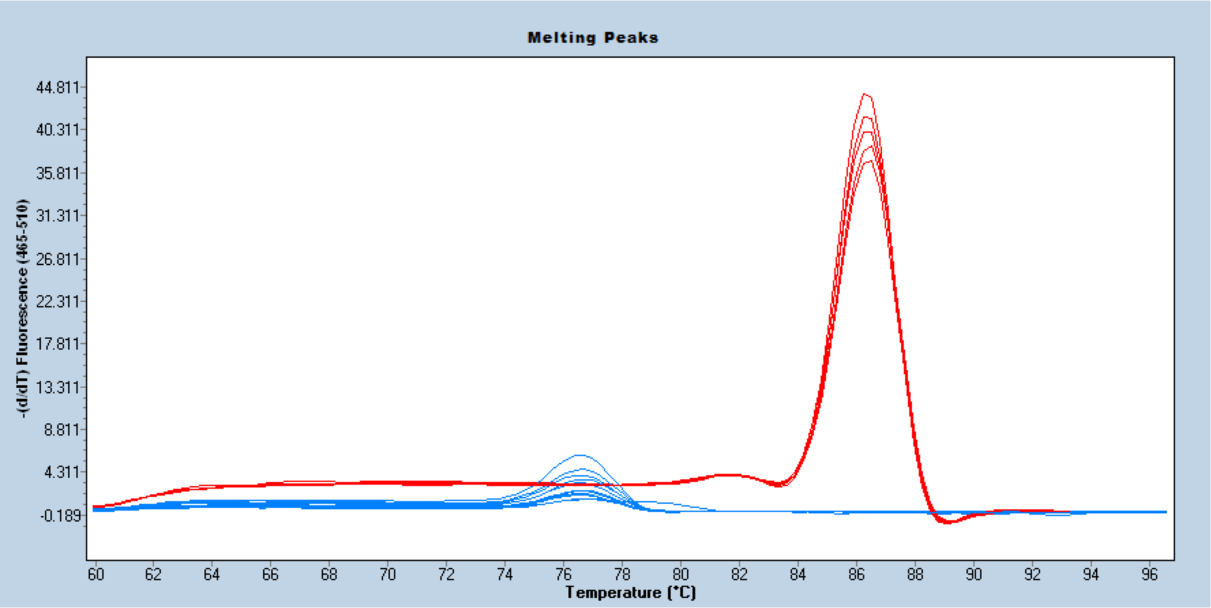

Figure S16. An example of picture showing melting peaks of the real-time PCR product specificity for the *bla*$_{OXA-23}$ gene – LightCycler 480 II (Roche, Basel, Switzerland); red lines – positive results, including positive control, showing specificity of the amplified product; blue lines – negative results.
